# Yields and Nutritional of Greenhouse Tomato in Response to Different Soil Aeration Volume at two depths of Subsurface drip irrigation

**DOI:** 10.1038/srep39307

**Published:** 2016-12-20

**Authors:** Yuan Li, Wenquan Niu, Miles Dyck, Jingwei Wang, Xiaoyang Zou

**Affiliations:** 1 Institute of Soil and Water Conservation, Northwest A&F University, Yangling 712100, Shaanxi, China; 2 Institute of Soil and Water Conservation, Chinese Academy of Sciences & Ministry of Water Resources, Yangling 712100, Shaanxi, China; 3 Institute of Water-saving Agriculture in Arid Areas of China, Northwest A&F University, Yangling 712100, Shaanxi, China; 4 Department of Renewable Resources, University of Alberta, Edmonton T6G2H1, AB Canada

## Abstract

This study investigated the effects of 4 aeration levels (varied by injection of air to the soil through subsurface irrigation lines) at two subsurface irrigation line depths (15 and 40 cm) on plant growth, yield and nutritional quality of greenhouse tomato. In all experiments, fruit number, width and length, yield, vitamin C, lycopene and sugar/acid ratio of tomato markedly increased in response to the aeration treatments. Vitamin C, lycopene, and sugar/acid ratio increased by 41%, 2%, and 43%, respectively, in the 1.5 times standard aeration volume compared with the no-aeration treatment. An interaction between aeration level and depth of irrigation line was also observed with yield, fruit number, fruit length, vitamin C and sugar/acid ratio of greenhouse tomato increasing at each aeration level when irrigation lines were placed at 40 cm depth. However, when the irrigation lines were 15 cm deep, the trend of total fruit yields, fruit width, fruit length and sugar/acid ratio first increased and then decreased with increasing aeration level. Total soluble solids and titrable acid decreased with increasing aeration level both at 15 and 40 cm irrigation line placement. When all of the quality factors, yields and economic benefit are considered together, the combination of 40 cm line depth and “standard” aeration level was the optimum combination.

Tomato (*Lycopersicon esculentum* Mill.) is one of the world’s main vegetable crops, and it is cultivated worldwide for fresh vegetable consumption or for processing. The Food and Agriculture Organization estimated 4.7 million cultivated ha of tomato worldwide in 2012 yielding 161 million metric tons, led by China producing 29.8% of this total^1^. Tomato is known as an important source of antioxidants such as vitamin C and lycopene in the human diet^2^,^3^, which have been linked with reduced risk of cancer, prostate, and heart diseases^3^,^4^,^5^,^6^. It is one of the most important vegetable crops in terms of production and acreage in both open-field and greenhouse production in northwest China. In recent years, tomato has quickly become one of the major vegetables grown in solar greenhouses in China because of its high potential yield and profitability.

Tomato quality depends on a combination of the interactions among different single quality attributes. It includes appearance (color, size, shape, lack of defects), flavor (total soluble solids, sugar, organic acid), nutritional value (lycopene, vitamin C, minerals) and storage qualities^7^,^8^. With the development of the social economy and the improvement of people’s living conditions in China, consumer demand is gradually shifting to higher quality tomatoes instead of quantity and, therefore, fruit quality should be considered in addition to yield.

Irrigation is the main way to supply water for crop growth in the greenhouse. In recent years, drip irrigation, sprinkler irrigation and subsurface drip irrigation (SDI), have been used in greenhouses in China^9^,^10^, and drip irrigation has been shown to be an effective method for high fruit yields in the greenhouse. It has been shown that SDI achieves higher production and water use efficiency than other irrigation methods^11^,^12^. Compared with furrow irrigation and sprinkler irrigation, SDI reduces weed germination and growth particularly in the area between rows^11^, and simultaneously increases fruit number and fruit size of tomato (yield), fruit quality and, therefore, increases water use efficiency and economic profits^9^,^12^,^13^.

Oxygen is essential for root respiration. It is well known that most plant roots require an adequate and continuous supply of soil O_2_ in order to respire, grow, develop, and function normally. This supply is obtained directly from the soil air. The relationship between soil moisture regime (influenced by both irrigation and rainfall) and tomato quality has been widely recognized^14^. However, irrigation increases the soil water content surrounding the roots, reducing air-filled porosity, and SDI is no exception. During and following irrigation events, as wetting fronts develop near the emitters, the root zone of the crop remains nearly saturated during and for some time after irrigation events, reducing availability and mobility of oxygen that remains trapped in soil pores, resulting in poor soil aeration in the crop root-zone, especially in heavy clay soils, or following excessive irrigation^15^,^16^. Compacted soils are also known to lack sufficient oxygen to sustain root activities^17^ Plant roots are sensitive to O_2_ deficiency which reduces root activities and plant performance in many species including cucumber and tomato^18^,^19^. Also, hypoxia in plant tissues decreases the production of ATP. This has many adverse effects including impacts on the H^+^-ATPases responsible for membrane function-membrane depolarization, for example^20^,^21^. In most crop plants, oxygen deprivation may cause severe injury, reduction of chlorophyll content, stomatal conductance, photosynthesis and transpiration rate of leaves^22^,^23^. Tomato plants are one of the most vulnerable mesophytes to hypoxia in the root environment^24^,^25^,^26^. Previous studies have shown that aerated irrigation enhances yield and quality of muskmelon in the greenhouse^26^; tomato roots are similar to muskmelon. Therefore, we hypothesize that anoxic conditions induced by SDI will be damaging to tomato crops also. With respect to soil aeration, irrigation results in soil oxygen decline and plant growth inhibition, which may be more acute in the greenhouse because the trafficking frequency of soil in a greenhouse is much higher than that of field soil. Wang *et al*.^27^ found that the average bulk density of subsurface soil (16–30 cm deep) in a greenhouse increases with increasing time of cultivation.

As early as the 1940s, Melsted *et al*.^28^ used Hydrogen peroxide as an oxygen fertilizer to improve oxygen concentration in root zone soil. Soil aeration can effectively improve nitrogen content of beans^29^. Increasing soil aeration has been found to be very useful in overcoming problems associated with hypoxia in the root zone of irrigated crops including tomato, Cucumber, cotton, zucchini and vegetable soybean, over a range of soil water contents and soil types, and it improves the performance of crops under oxygen-deficient conditions^30^,^31^,^32^,^33^,^34^,^35^. Usually, air pumps, super micro bubble generating systems or venturi injectors are used to aerate irrigation water. These methods are also named subsurface oxygation, supplemental soil aeration or aerated irrigation^32^,^33^,^36^. The air injected into the irrigation lines via a manifold connected to an air compressor was previously demonstrated by Li *et al*.^30^,^37^. Li *et al*.^30^ found that injection of air to the soil through subsurface trickled irrigation tubes during 5 different periods significantly enhanced the growth of root and fruit in potted tomato plants.

It was hypothesized that varying the volume of air pumped into the root zone and burial depths of drip irrigation tubes (aeration position) would result in an improved soil air environment in the root zone, increase microbial abundance, soil enzyme activity, total length and surface area of roots, and promote nutrient uptake, thus promoting plant growth and fruit output^36^,^38^. To date, there are no reports in the literature specifically examining the sensitivity of tomato plants in clay loam to soil aeration volume and burial depths of drip irrigation tubes, and how this may impact plant growth and fruit quality. However, such information would have high practical value, and this is especially important in greenhouse.

Tomato is a moderate-rooted plant- the roots are mainly distributed within 40 cm of the soil surface. If aeration lines (irrigation lines) are placed at a shallow depth (10 cm), plant growth and fruit yield might not be improved significantly because of the chimney effect. However, if placed at greater depths (50 cm), placing is much more labor-intensive. Synthesizes each kind of situation, the experiment used 15 and 40 cm horizontal drip irrigation line burial depths. Therefore, the objective of this study was to quantify effects of non-aeration versus 3 aeration levels in greenhouse tomato plants at 2 depths of drip irrigation tube placement. Specifically, we examined data on plant growth, output and nutritional value of the fruits. The best depth and aeration treatment combinations are acquired for the improvement the comprehensive benefit of tomato, which may provide to tomato production.

## Materials and Methods

### Experimental site and soil details

The experiments were conducted in a greenhouse near Yangling (latitude34°17′N, longitude108°02′E, altitude520 m), Shaanxi Province of northwest China between October 18, 2014 and May 20, 2015. The site has an average annual sunshine of 2163.8 h, and 114.8 kCal/cm^2^ of annual total amount of radiation. The bulk density of the soil was 1.34 g·cm^−3^, field capacity was 28.17% (moisture content by mass), pH was 7.82, and soil porosity was 49.38%. Sand (2–0.02 mm) accounted for 25.4% of the soil, silt (0.02–0.002 mm) accounted for 44.1%, and clay (<0.002 mm) accounted for 30.5%.

### Experimental design and Treatments

**Figure 1:**
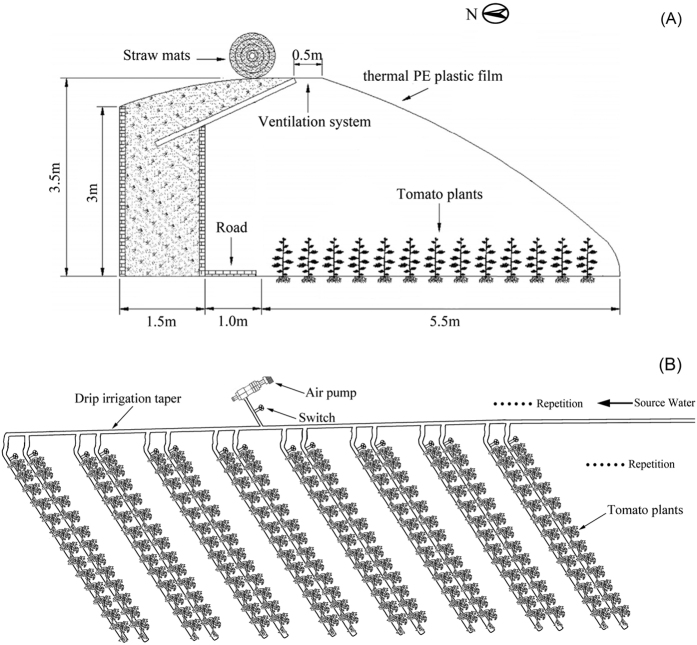
(**A**) Cross-sectional of the greenhouse in Northwest china; (**B**) Experimental arrangement of an example block. Treatments were randomized within each block.

The greenhouse was 108 m long and 8 m wide with an east-west orientation, while each cultivation plot was 5.5 m long and 1.5 m wide with total planting area of 8.25 m^2^, and crop rows were aligned north-south. The greenhouse had no temperature control system. In order to maintain the interior temperature at night during the winter, straw mats were spread on the surface of the thermal PE plastic film; and during the daytime, the interior temperature was controlled by a ventilation system on the roof (Fig. 1A).

Two subsurface drip irrigation lines with diameters of 16 mm were laid down in each cultivation area; the spacing between the drippers was 30 cm, and the spacing between the drip irrigation tubes was 0.5 m. Both water and air were supplied to the soil through the main irrigation pipe and subsurface drip irrigation lines. The row spacing for planting was 0.5 m in each plot, the planting depth was 0.2 m, and the plant spacing was 0.4 m. Two rows were planted in each area, with 13 plants in each row. To prevent the lateral spread of air and water into adjacent treatments, the plots were separated from each other by a 1.5 m wide empty space (Fig. 1B). 3-week old tomato seedlings of variety “Fen-yu-Yang-gang” were transplanted and the preceding crop cultivated in the greenhouse was muskmelon. The gas for soil aeration was air, and the soil for the test was a Lou silty clay loam (classified as Inceptisol according to USDA soil taxonomy).

The experimental design was treated as a 2 × 4 full-factorial design with 3 replicates for statistical analyses. The experiment used two horizontal drip irrigation line burial depths (D); and these tubes were used for both for irrigation and aeration. Treatments are abbreviated as: D15 and D40 representing drip irrigation tube burial depths of 15 cm and 40 cm, respectively, and aeration volumes (V), CK, V1, V2 and V3 represent no aeration and 0.5, 1 and 1.5 times the standard aeration volume, respectively. The standard aeration volume was calculated according to *V* = *S *× *L *× *porosity*^39^, where *V* is the volume (L) of each aeration plot, *S* is the cross-sectional area (1,500 cm^2^) of the ridge, *L* is the length of the ridge (550 cm). Accordingly, the calculated standard aeration volume was 407.83 L. In all experiments that varied the aeration volume, the aeration frequency was once every two days. Three air pumps supplied air equally to the main drip irrigation line. The air discharge rate of the drippers was about 0.28 L/min. Aeration was carried out once every two days from 4 to 6 PM during the whole growing season (1–214 days after transplant).

The soil fertility in this area is moderate. All agronomic management measures taken during growth period of tomato such as fertilization, agricultural chemicals spraying, etc. were consistent with local production practices. Before transplanting, the soil was rototilled, and 120 t ha^−1^ of decomposed organic manure (pig and sheep manure), 1500 kg ha^−1^ of diammonium phosphate (N 18% and P_2_O_5_ 46%) and 400 kg ha^−1^ of compound fertilizer (N 18%, P_2_O_5_ 15%, and K_2_O 12%) were broadcast uniformly as the basal fertilizer in the soil. The average total amount of irrigation was 235 mm. Irrigation water demand is estimated mainly by farmers’ perceptions and the climatic conditions. The average total amount of irrigation applied was 235 mm. Young tomato fruits with the same pollination date, same node, and similar fruit size were marked in each treatment.

### Measurements

To observe the dynamic change of plant growth, plant height was measured using a steel ruler before the main tips were cut, and the diameter of the plant stem was measured by a digital Vernier caliper.

For the first fruit picking, three fruits per treatment were sampled for measurement among the marked fruits. After weighing, the fruit sample was measured for width (equatorial) and length (polar), fruit diameter of each fruit (mm) using a Vernier caliper, and then the flesh samples (skin and seeds removed) were juiced with a domestic juicer, and the juice was decanted and subjected to a series of tests for the following quality parameters: vitamin C, lycopene, total soluble solids and titrable acid. Vitamin C content was determined by molybdenum blue colorimetry^40^,^41^. This method is based on the reaction of ascorbic acid (VC) with ammonium phospho-molybdate in the presence of SO_4_^2−^ and PO_4_^3−^, generating blue molybdenum. This blue molybdenum has maximum light absorption at a wave length of 760 nm. This vitamin C assay method is accurate, repeatable, and insensitive to the interference in the presence of common reducing sugars. Lycopene was extracted with 2% dichloromethane and petroleum as solvents to enhance the solubility of lycopene, and the absorption at 502 nm was subsequently measured^42^. The taste and nutritional properties of the first ripe fruit harvested from the first truss of each tagged plant were determined. The fruit was sliced and blended after removing the skin and seeds. A hand-held ATAGO-P32 temperature compensated refractometer (ATAGO Co. Ltd, Tokyo, Japan) was used to directly read the % soluble solids (as ^o^Brix) of the blended fruit at room temperature^43^. Titratable acids (% by weight) were determined by diluting an aliquot of the blended fruit and titrating against 0.1 M NaOH using Phenolphthalein as an indicator. The % by weight titratable acids was estimated as (ml NaOH x acid factor = 0.0064) divided by ml aliquot of blended fruit^44^,^45^.

### Calculation of irrigation-use efficiency

The total volume of irrigation water applied was recorded. It was assumed that this volume was uniformly distributed over all of the plots. Therefore, the irrigation volume for each treatment was estimated as the total irrigation water volume divided by 8. The observed weight of tomato fruits harvested from each treatment for a given treatment was summed to obtain the total number of fruits. The observed total yield of tomato from each treatment was calculated by adding together first picking and second picking of fruits. Irrigation water use efficiency (IWUE, kg/m^3^), defined as the ratio of fruit yield (kg) to the seasonal amount of irrigation water applied per plot, was calculated as the IWUE = (*Y*/*I*), where *Y* is the total yield (kg· plot) and *I* is the amount of applied water (m^3^·plot) for each treatment.

### Statistical analysis

The experimental data were analysed using the two-way ANOVA procedure in SPSS Statistics 22.0 (IBM Crop., Armonk, New York, NY, USA), and the differences were compared using the Duncan’s test with a significance level of 0.05.

All figures were constructed using the graphing software Origin-Pro 8.5 (Origin Lab Corporation, Northampton, MA, USA) and Photoshop CS 5 (Adobe Systems Inc., San Jose, California, USA).

## Results

### Plant growth characteristics of different treatments

The cumulative trends of the vegetative growth parameters (stem diameter and plant height) at 25, 46, 65, 73, 82 and 96 days after transplant (DAT) for 4 aeration levels and 2 depths of drip irrigation tubes are presented in Table 1. Stem diameter and plant height were measured before tip pruning, during the vegetative growth stage from 12 November 2014 to 22 January 2015.

The results showed the root zone aeration had a significant effect on plant height during 25 to 73 DAT and 46, 96 DAT of stem diameter. Emitter depth both had a significant effect on plant height and stem diameter at 82 DAT. The ANOVA F-value showed the interaction of irrigation line depth and aeration level is significant for plant height at 25, 46 and 73 DAT but not at 65, 82 and 96 DAT for plant height and stem diameter (Table 1). Even though the root zone aeration treatments did not significantly impact (P > 0.05) the plant height during 82 to 96 DAT, mean plant height numerically higher with the increasing of aeration volume. Different burial depths of drip irrigation tubes had significant impacts on plant height at 65 DAT in the V3 treatment, and had significant impacts on stem diameter at 82 DAT in the CK and V2 treatments.

### The effect of different treatments on the yield and related production functions of tomato

**Figure 2:**
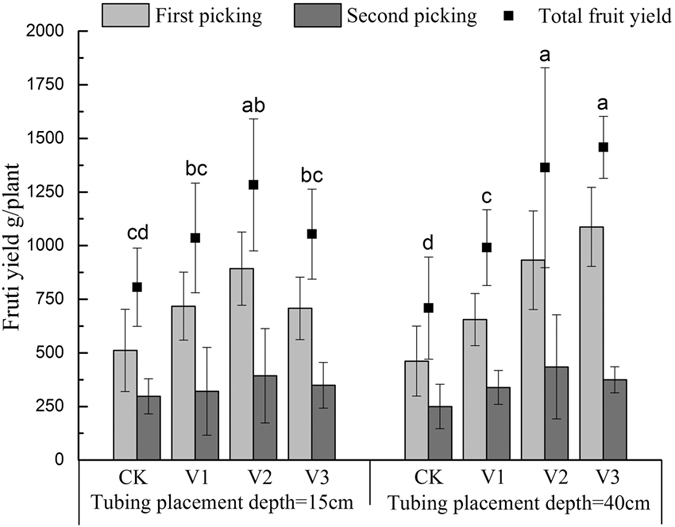
Fruit yield (g/plant) at 150 days after transplant (first picking, n = 9) and 205 days after transplant (second picking, n = 9) from tomato plants for 4 aeration treatments (i.e. none or aeration applied for 3 different volumes). Data are the means of nine replicates. with standard deviations shown by vertical bars. Different letters are significantly different between the treatments at 0.05 level according to Duncan’s test.

Figure 2 shows the yield of greenhouse tomatoes under different aeration treatments at 2 depths of drip irrigation lines. It can be seen that in each depth of drip irrigation line aeration treatments had significantly higher total yield than that of no-aeration, which indicates that tomato yields were sensitive to root zone aeration. First and second picking yields and total fruit yields showed first an increase and then a decrease with the increase of aeration volume at 15 cm irrigation line depth. It was clear that at 15 cm line depth, tomato yield in the D15V2 treatment was higher than that of other aeration treatments. It can also be seen that the first picking fruit weight and total fruit weight at the 40 cm line depth increased with the increase of aeration volume. D40V3 and D40V2 treatment had the highest total production.

**Figure 3:**
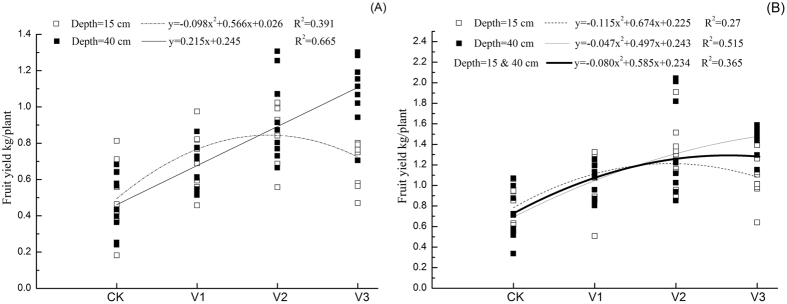
The relationship between artificial soil aeration volume with fruit yield of first picking (**A**), and total fruit yield (**B**).

Figure 3 summarizes the response first picking fruit yield (A) and total fruit yield (B) to aeration. First picking and total yields responded to higher levels of aeration in the 40 cm irrigation depth treatment, but yields tended to decrease at higher levels of aeration at the 15 cm line depth.

### The effect of different treatments on tomato shape and IWUE

The ANOVA showed significant main treatment effects on fruit yields, mean fruit weight, fruit width, fruit length and IWUE but not on fruit number (Table 2). Aeration volume had a significant effect on the fruit yield, fruit length and IWUE. The aeration volume × line depth interaction was significant in mean fruit weight and fruit width whereas it was non-significant for fruit yield, fruit number, fruit length and IWUE.

Total fruit yields increased from 23.96 to 39.68 t/ha when the scheduled aeration changed from CK to the V2 and V3 levels, with V2D40 and V3D40 having the maximum fruit yields and CKD40 having the minimum observed fruit yield.

There was no significant difference on fruit number under different treatments (Table 2). Mean fruit weight and fruit width were the highest in treatment V1D15, whereas fruit length reached the maximum in the V3D40 treatment.

IWUE for different treatments is listed in Table 2. The results showed that tomato IWUE showed an initial increase followed by a decrease, in the order of V2 > V3 > V1 > CK when the irrigation line depth was 15 cm. For the 40 cm line depth, the V2 and V3 aeration treatments showed the highest values of IWUE.

**Figure 4:**
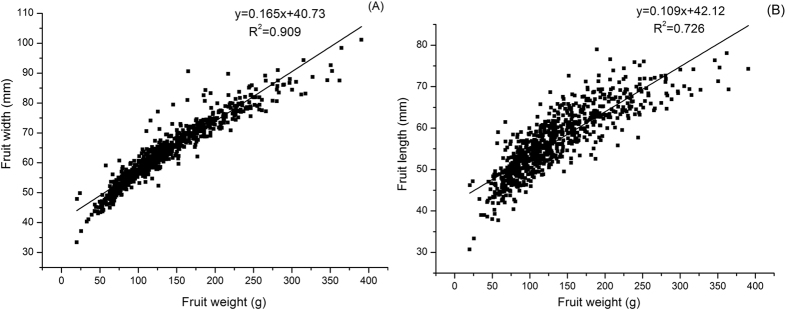
The relationship between fruit weight with fruit width (**A**) and fruit length (**B**).

Figure 4 shows the relationship between fruit weight and fruit width (A) and fruit length (B). From Fig. 4(A) and (B), it can be seen that fruit width and fruit length which showed the fruit shape is important for fruit weight. Figure 4(A) shows a highly significant determination factor (R^2^ = 0.909) for the relations of tomato fruit yield with fruit width in during the aeration treatments.

### The effect of different treatments on tomato quality

Vitamin C and lycopene content of tomato fruits in all treatments are presented in Table 3. Variation in soil aeration and depth of irrigation line had no significant effects on vitamin C content, but a positive relationship between vitamin C content and increasing volume of aeration was observed.

It appears that soil aeration had positive effects on lycopene content of tomato (Table 3). The results showed that lycopene contents under all the aeration treatments were significantly higher than with no-aeration. Lycopene showed an initial increase followed by a decrease when the aeration volume was increased at both 15 and 40 cm irrigation tubes in CK and V3.

Table 4 shows total soluble solids, titrable acid and sugar-to-acid ratio under different aeration volume and depth of tubing. At each burial depth of drip irrigation tubes, aeration volume can significantly affect total soluble solids and Sugar-to-acid ratio (P < 0.01). Also, at the 15 cm irrigation line depth, aeration volume significantly affected the titrable acid (P < 0.01). The interaction between aeration volume and the burial depth of drip irrigation line had an extremely significant effect on total soluble solids and sugar-acid ratio (P < 0.01), and significant effect on titrable acid (P < 0.05).

As can be seen in Table 4, at the 15 cm line depth, total soluble solids decreased with increasing aeration volume. At the 40 cm line depth, total soluble solids decreased with increasing aeration volume from CK to V2, however total soluble solids reached the maximum observed value in theV3 treatment.

The effects of different combinations of aeration volume and irrigation line depth on titrable acid are shown in Table 4. Titrable acid was highest in CK treatment at both 15 and 40 cm line depths. As can be seen in Table 4, titrable acid decreased with the increasing aeration volume for CK to V3 from 0.37% to 0.22% at the 40 cm line depth. Nevertheless, at the 15 cm depth, titrable acid increased with increasing aeration volume from V1 to V3, corresponding to a 15.6% increase from 0.32% to 0.37%.

As would be expected, the responses of sugar-to-acid ratio to aeration volume showed a similar pattern to vitamin C. Also sugar-to-acid ratio increased with the increase of aeration volume at 40 cm line depth. And the sugar-to-acid ratio decreased with the increasing aeration volume for V1 to V3 from 18.64 to 12.02 at 15 cm depth of tubing. The highest sugar/acid ratio was obtained at D15V1 and D40V3 treatment combinations.

Table 5 shows that the correlation between vitamin C, lycopene, total soluble solids, titrable acid and sugar-to-acid ratio of tomato. Only sugar-to-acid ratio showed a significant positive correlation with total soluble solids (P < 0.01). Titrable acid showed an extremely significant negative correlation with sugar-acid ratio (P < 0.01). The vitamin C and lycopene did not show a significant correlation with other quality parameters.

## Discussion

Previous research shows using SDI to provide aerated water can effectively enhance performance of the crops^31^. Our results showed that both aeration volume and depths of drip irrigation lines influenced plant growth, fruit yield and shape. Fruit quality (i.e., Vitamin C and lycopene) was also enhanced by aeration treatment.

### Plant growth and fruit yield

As reported in a previous study, hypoxia stress resulted in increased phytohormones such as ABA, ethanol and ethylene^23^,^46^,^47^. ABA is an important chemical signal which leads to stomatal closure^48^. The accumulation of ABA was shown to lead to stomatal closure, reduced stomatal density and decreased net photosynthesis rate^49^. Hypoxia induces a shift from normal respiration to anaerobic respiration and fermentative production of ethanol and lactic acid^49^. Fukao’s research^50^ suggested that the hypoxia stress in crops decreased the transportation of oxidative phosphorylation electrons, which decreased the generation of ATP and NADP (H)^+^.

Our results showed that soil aeration had a positive effect on both plant height and stem diameter (Table 1), suggesting that hypoxia stress under non-aerated conditions in greenhouse tomato in this soil causes reduced production, and the applied soil aeration treatments alleviated root zone hypoxia. Our studies also reveal reduced influence of aeration on plant height at the later stages of the experiment (82, 96 DAT). This result is likely because soil aeration affects tomato plants differently at different growth stages, and this is consistent with previous observations^30^,^51^. Further, this result is also consistent with previous studies that observed aeration hastened flowering in soybean and cotton in pot trials^31^, and there was an indirect indication of earlier maturity of pumpkin in another field trial^31^,^52^. Aeration and irrigation line depth were found to markedly affect the yields of the tomato crops (Fig. 2), particularly at 150 DAT (first picking), probably because different aeration and irrigation depth (position) would result in different soil air and water redistribution. Bhattarai *et al*.^33^ also reported an enhanced the first inflorescence due to aeration treatment in heavy clay and saline soils compared with non-aeration.

Maximum observed total fruit yields were in the D15V2, D40V2 and D40V3 treatments Similarly, it can be seen from regression analysis, increasing aeration volume resulted in higher total fresh fruit yield at 40 cm line depth, but under the 15 cm line depth treatments yields first increased and then decreased with increasing aeration. These results suggest that soil aeration is no longer a limiting factor after a certain level for the shallow irrigation treatments. Once aeration had already eliminated hypoxic stress in the soil, the more air injected may not have resulted in reduced hypoxia, but escapes the soil to the atmosphere which resulted in a reduction in root-to-soil contact which had a negative effect on the rhizosphere environment, and decreased fruit yields. With irrigation tubes placed at 40 cm, however, the location of tubes is below the major of plant rooting zone and the positive effect of aeration (alleviate hypoxia) on plant roots will gradually appear only with high aeration volumes.

### Tomato shape and quality

Tomato is considered an important commercial vegetable. Visual appearance is an important factor driving the initial consumer’s choice. Our data suggests that aeration treatments also had a positive impact on width and length of fruit. As expected, the mean fruit width and length for all the aeration treatments were higher than with no-aeration.

In addition to the effects on fruit appearance, aeration is known to influence the quality and taste^26^,^53^. Our previous studies had shown that soil aeration at different growth stages lead to higher quality of potted tomatos^30^. Consistent with Bhattarai *et al*.^53^, significantly greater total soluble solid cucurbits were reported in the aeration treatments. Horchani *et al*., suggested that prolonged root hypoxia of tomato could significantly limit ascorbate accumulation during fruit ripening, and suggested that the primary mechanism that limits the ascorbate accumulation in fruits is based on a reduced induction of most of the genes in their biosynthesis pathways^24^. To date, there is scarce information about the aeration volume combined with burial depths of drip irrigation tubes on tomato quality in greenhouse. In this study, the aeration led to an increase in vitamin C, lycopene, and Sugar/acid ratio by 41%, 2%, and 43%, respectively, in the V3 compared with the no- aeration.

### Economic analysis and evaluation

Based on the local labor force price (female workers = 50 yuan/day, male workers = 100 yuan/day), preparation of 15 and 40 cm line depths for a whole greenhouse was 200 yuan and 600 yuan, respectively. The labor price of soil aeration per hour was 50/8 = 6.25 yuan. And the labor cost of aeration for V1, V2 and V3 throughout the whole growing season was 892, 1783 and 2675 yuan, respectively. Additional labor costs included ditching costs and operation of the aeration pump and monitoring costs. The additional labor cost and electricity cost obtained in the different treatments is shown in Table 6.

Because the spacing between each plot was quite large, total fruit yield for each treatment was lower than the normal cultivation patterns. The tomato price can vary greatly from year to year and the average price for several years was 4.5 yuan/kg. In addition, the tomato price plays an important role in the gross income every year for local farmers. Additional income for each treatment is shown in Table 6 and all of the aeration treatments at 40 cm line placement depth and the V1 and V2 aeration treatments with 15 cm line depth resulted in greater income. The calculated maximum total income was 12312 yuan per greenhouse for the D40V3 treatment combination. Results showed that the comprehensive benefit order for every treatment combination was D40V2 > D15V2 > D40V3 > D15V1 > D40V1 > D15CK > D40CK > D15V3. Soil Aeration could improve yield of greenhouse tomato differently, but the comprehensive benefit of some aeration treatments decreased because of the investment of additional labor, and electricity.

Nevertheless, the calculation does not consider the influence of fruit quality, and soil aeration has positive effects on vitamin C, lycopene content and sugar/acid ratio of tomato but these factors don’t currently affect tomato prices and, therefore, their economic benefit can’t be estimated.

### Optimum treatment combination for greenhouse tomato in Lou soil

Our results show that both 15 and 40 cm line depths can be applied to greenhouse tomato. The comparison of different burial depths with the same aeration conditions found that the yields were greater with a 15 cm irrigation line burial depth than with 40 cm for the CK and V1 treatments, but yield was higher for 40 cm burial depths compared to the 15 cm deep burial with the other treatments. When the burial is shallow, excessive aeration volume (V3 and V4) was associated with decreasing tomato yield. This phenomenon may occur because at 15 cm soil depth, the tomato roots are abundant, the position of air application is in the main root area of the plant, and aeration can alleviate hypoxic stress. However, a large amount of aeration increases the disturbance to the soil and increasing cavitation at the root area (a reduction in root-to-soil contact) and that can have a negative effect on plant roots. In contrast, the pattern is exactly opposite when the drip irrigation tubes are buried at 40 cm. In this case, the air uses the soil medium as the buffer, the main rooting depths are above the aeration position, and the effect of the air on the roots is not as direct as with a 15 cm deep tube burial. The airflow in the root area is much slower than with a 15 cm deep burial condition. At the same time, aeration largely relieves the hypoxic stress in the root area. Thus, with a 40 cm deep burial, large volume of a aeration can increase the plant yield more significantly.

## Conclusions

Our results indicated that the different soil aeration levels and depths of drip irrigation tubes had significant influence on tomato’s yield and nutritional quality with SDI grown in a greenhouse. Soil aeration enhanced greenhouse tomato plant growth, yield fruit shape and nutritional quality. These parameters generally increased with increasing aeration when irrigation tubes were placed at 40 cm, but increased and then decreased with increasing aeration volumes when tubes were placed at 15 cm depth. While D40V2 and D40V3 treatment combinations had the highest fruit yield because of the better efficiency with soil aeration, the highest economic benefits were obtained from D40V2 treatment. The results suggested that aeration can alleviate temporal hypoxia associated with drip irrigated tomato crops in Lou soil and also offer yield and fruit quality benefits.

## Additional Information

**How to cite this article**: Li, Y. *et al*. Yields and Nutritional of Greenhouse Tomato in Response to Different Soil Aeration Volume at two depths of Subsurface drip irrigation. *Sci. Rep.* **6**, 39307; doi: 10.1038/srep39307 (2016).

**Publisher's note:** Springer Nature remains neutral with regard to jurisdictional claims in published maps and institutional affiliations.

## Figures and Tables

**Table 1:** Mean plant height and stem diameter for different treatments during vegetative growth period.

	Days after transplant	CK			V1			V2			V3			F-value		
		D15	D40	T-test	D15	D40	T-test	D15	D40	T-test	D15	D40	T-test	V	D	V*D
Plant height (cm)	25	36.44 ± 2.40c	36.22 ± 2.59c	ns	38.33 ± 1.66bc	37.67 ± 1.66bc	ns	41.00 ± 2.65a	37.56 ± 2.13bc	**	40.11 ± 3.14ab	41.44 ± 3.36a	ns	**	ns	*
	46	58.33 ± 2.18bc	51.11 ± 2.03c	**	53.89 ± 7.27bc	54.78 ± 2.99bc	ns	54.33 ± 19.31bc	61.00 ± 3.04ab	ns	69.00 ± 9.37a	62.33 ± 5.27ab	ns	**	ns	*
	65	77.22 ± 8.94 cd	75.33 ± 6.75d	ns	83.11 ± 6.01abc	81.89 ± 3.52abc	ns	86.56 ± 6.39a	84.00 ± 7.30ab	ns	86.67 ± 3.00a	78.44 ± 4.28bcd	**	**	*	ns
	73	78.44 ± 8.88d	91.89 ± 4.86c	**	92.33 ± 3.91bc	89.00 ± 2.24c	*	92.78 ± 5.04bc	93.11 ± 2.98bc	ns	99.67 ± 2.55a	97.11 ± 5.35ab	ns	**	ns	**
	82	102.22 ± 11.40abc	95.22 ± 7.05c	ns	101.67 ± 5.70abc	97.00 ± 3.46bc	ns	106.56 ± 9.11a	100.22 ± 4.32abc	ns	103.56 ± 5.64ab	102.67 ± 7.25abc	ns	ns	**	ns
	96	114.44 ± 11.49a	114.33 ± 4.85a	ns	113.44 ± 7.23a	114.44 ± 9.37a	ns	117.00 ± 9.60a	109.67 ± 5.63a	ns	110.89 ± 9.09a	114.22 ± 9.42a	ns	ns	ns	ns
Stem diameter (mm)	25	6.91 ± 1.47bc	6.82 ± 1.05c	ns	7.49 ± 2.19abc	7.69 ± 0.84abc	ns	7.28 ± 0.61abc	8.29 ± 0.48ab	**	7.87 ± 1.51abc	8.35 ± 0.51a	ns	ns	ns	ns
	46	7.17 ± 0.66c	7.02 ± 1.41c	ns	7.48 ± 0.62bc	7.37 ± 1.06bc	ns	8.54 ± 0.89ab	8.87 ± 1.53a	ns	9.36 ± 1.26a	9.27 ± 1.64a	ns	**	ns	ns
	65	9.21 ± 0.97ab	7.56 ± 1.68b	*	10.07 ± 3.27ab	8.14 ± 3.00b	ns	10.07 ± 2.84ab	9.25 ± 2.12ab	ns	9.29 ± 2.40ab	11.39 ± 2.12a	ns	ns	ns	ns
	73	10.35 ± 0.52ab	7.80 ± 1.32b	**	10.61 ± 4.08ab	9.75 ± 3.15ab	ns	9.71 ± 2.62ab	10.54 ± 1.08ab	ns	10.98 ± 3.11a	11.73 ± 3.30a	ns	ns	ns	ns
	82	9.63 ± 1.62b	11.53 ± 0.84ab	**	10.46 ± 1.90ab	11.80 ± 2.33a	ns	10.63 ± 1.39ab	12.35 ± 1.99a	*	11.25 ± 2.39ab	11.85 ± 1.87a	ns	ns	**	ns
	96	8.95 ± 2.03b	8.83 ± 1.39b	ns	10.56 ± 1.49ab	9.72 ± 1.16ab	ns	9.70 ± 0.97ab	10.95 ± 2.51a	ns	10.66 ± 2.18ab	11.43 ± 1.90a	ns	**	ns	ns

Data were shown in mean ± standard deviation (n = 9). The values with the same letter within rows are statistically non-significant by Duncan’s test at p < 0.05. The t-test was used to compare 2 depths of drip irrigation tubes (n = 9) for each aeration treatment. The asterisk indicates significantly different irrigation means (^*^for ≤0.05, ^**^for ≤0.01), otherwise not significant (ns). ANOVA F-value for main and interaction effects were not significant (ns) or significant at ≤0.05 (*) and ≤0.01 level (**).

**Table 2:** Some parameters of yield and IWUE under different aeration treatments.

Parameters	Treatments										
	CK		V1		V2		V3		F-value		
	D15	D40	D15	D40	D15	D40	D15	D40	V	D	V*D
Total fruit yield (t/ha)	25.49bc	22.43c	32.74abc	31.32abc	40.53ab	43.07a	33.30abc	46.06a	5.989**	0.666 ns	1.148 ns
Fruit number	24a	20a	22a	21a	24a	24a	24a	26a	0.589 ns	0.040 ns	0.355 ns
Mean fruit weight (g)	133.88ab	124.90b	155.30a	128.85b	120.68b	141.16ab	128.28b	136.50ab	.988 ns	0.096 ns	3.348*
Fruit width (mm)	60.06c	61.09bc	66.93a	62.06bc	62.50bc	64.82ab	61.86bc	63.74abc	2.135 ns	0.105 ns	2.983*
Fruit length (mm)	55.72b	55.13b	56.81ab	56.98ab	58.56ab	56.41ab	56.35ab	59.64a	2.709*	0.176 ns	1.701 ns
IWUE (kg/m^3^)	15.45bc	13.59c	19.84abc	18.98abc	24.57ab	26.11a	20.18abc	27.91a	5.989**	0.667 ns	1.148 ns

The values with the same letter within rows are statistically non-significant by Duncan’s test at p < 0.05. ANOVA F-value for main and interaction effects were not significant (ns) or significant at ≤0.05 (^*^) and ≤0.01 level (^**^).

**Table 3:** Effects of different aeration treatments on Vitamin C and lycopene of tomato.

Treatments	Vitamin C (mg/100 g)				Lycopene content (ug/g)			
	D15	D40	T-test	Mean	D15	D40	T-test	Mean
CK	20.6 ± 1.8b	20.9 ± 2.2b	ns	20.8 ± 1.9a	32.77 ± 7.29b	42.64 ± 13.06b	ns	37.70 ± 11.45b
V1	24.3 ± 7.1ab	25.9 ± 4.0ab	ns	25.1 ± 5.6a	47.16 ± 17.94a	72.93 ± 21.42a	*	60.04 ± 23.31a
V2	26.5 ± 1.6ab	26.8 ± 4.4ab	ns	26.6 ± 3.2a	42.81 ± 12.57ab	60.73 ± 21.26ab	*	51.77 ± 19.29a
V3	28.5 ± 14.4a	30.1 ± 13.0a	ns	29.3 ± 13.4a	33.38 ± 4.82b	43.20 ± 21.44b	ns	38.29 ± 15.90b
*F*-value								
Aeration volume (V)	1.543 ns(D15)	2.483 ns(D40)			3.265*(D15)	5.033**(D40)		
emitter depth (D)	0.272 ns				17.242**			
Interaction (V × D)	0.048 ns				1.001 ns			

Data were shown in mean ± standard deviation (n = 9). Aeration treatment means at each depth of drip irrigation tube (n = 9) not followed by the same letter within columns are significantly different at the 0.05 level. The t-test was used to compare 2 depths of drip irrigation tubes (n = 9) for each aeration treatment. The asterisk indicates significantly different irrigation means (^*^for p ≤ 0.05, ^**^for p ≤ 0.01), otherwise not significant (ns). ANOVA F-value for main and interaction effects were not significant (ns) or significant at ≤0.05 (*) and ≤0.01 level (**).

**Table 4:** Effects of different aeration treatments on Total soluble solids, soluble sugar and titrable acid of tomato.

Treatments	Total soluble solids (%)				Titrable acid (weight %)				Sugar/acid ratio			
	D15	D40	T-test	Mean	D15	D40	T-test	Mean	D15	D40	T-test	Mean
CK	5.53 ± 0.09a	5.11 ± 0.20bc	**	5.32 ± 0.26a	0.52 ± 0.09a	0.37 ± 0.13a	*	0.44 ± 0.13a	11.05 ± 2.12b	15.48 ± 5.01b	*	13.27 ± 4.37b
V1	5.29 ± 0.13a	5.17 ± 0.48b	ns	5.23 ± 0.35a	0.32 ± 0.11b	0.32 ± 0.05ab	ns	0.32 ± 0.08b	18.64 ± 6.34a	16.66 ± 3.52b	ns	17.65 ± 5.08ab
V2	4.90 ± 0.41b	4.74 ± 0.20c	ns	4.82 ± 0.32b	0.36 ± 0.05b	0.29 ± 0.15ab	ns	0.33 ± 0.11b	13.70 ± 1.72b	20.56 ± 9.48ab	*	17.13 ± 7.50ab
V3	4.39 ± 0.26c	5.66 ± 0.54a	**	5.02 ± 0.77ab	0.37 ± 0.05b	0.22 ± 0.03b	**	0.30 ± 0.09b	12.02 ± 1.29b	25.83 ± 4.74a	**	18.93 ± 7.86a
*F*-value												
Aeration volume (V)	34.243**	8.349**			10.158**	3.077*			8.313**	5.223**		
Emitter depth (D)	3.336 ns				17.920**				24.128**			
Interaction (V × D)	24.122**				2.752*				7.685**			

Data were shown in mean ± standard deviation (n = 9). Aeration treatment means at each depth of drip irrigation tube (n = 9) not followed by the same letter are significantly different at the 0.05 level. The t-test was used to compare 2 depths of drip irrigation tubes (n = 9) for each aeration treatment. The asterisk indicates significantly different irrigation means (^*^for p ≤ 0.05, ^**^for p ≤ 0.01), otherwise not significant (ns). ANOVA F-value for main and interaction effects were not significant (ns) or significant at ≤0.05 (*) and ≤0.01 level (**).

**Table 5:** Correlation between Some quality parameters of tomato.

	Vitamin C	Lycopene	Total soluble solids	titrable acid	Sugar-acid ratio
Vitamin C	1				
Lycopene	0.046	1			
Total soluble solids	−0.044	−0.019	1		
titrable acid	−0.148	−0.135	−0.009	1	
Sugar-acid ratio	0.115	0.109	0.345**	−0.878**	1

^**^Significant at the 1% (2-tailed).

**Table 6:** Economic analysis for the soil aeration treatments in each greenhouse.

Treatments		Additional labor cost (yuan)	Additional electricity (yuan)	Depreciation of the air pump (yuan)	Total Yield (kg)	Total income (yuan)	Additional income compared with D15CK (yuan)
D15	CK	200.00	0.00	0.00	1514	6812	0
	V1	1091.67	40.13	200.00	1945	8752	807
	V2	1983.33	80.25	300.00	2408	10834	1858
	V3	2875.00	120.38	600.00	1978	8901	−1307
D40	CK	600.00	0.00	0.00	1332	5995	−1217
	V1	1491.67	40.13	200.00	1860	8372	28
	V2	2383.33	80.25	300.00	2559	11514	2138
	V3	3275.00	120.38	600.00	2736	12312	1704
